# The familiar dialectic between overclaiming and moral outrage over brain biology: disconnected from what matters

**DOI:** 10.1017/S0033291721003184

**Published:** 2021-12

**Authors:** Steven E. Hyman

**Affiliations:** Stanley Center for Psychiatric Research, Broad Institute of MIT and Harvard, Cambridge, MA 02142, USA

In his appraisal of American psychiatry, the historian Andrew Scull expresses well-warranted indignation at the puffery of some leading figures in organized psychiatry over the state of psychiatric diagnosis and progress in therapeutics. Scull vividly reports public disagreements that characterized the development of the Diagnostic and Statistical Manual of Mental Disorders, 5th edition (American Psychiatric Association, 2013) that in some cases were driven as much by wounded narcissism as by science (Scull, 2021). Ultimately, however, the analysis promised by Scull's ambitious title does not land. Accounts of inflated promises and deflating rebuttals traded in rarified circles (including some of my own characteristically unrestrained statements to the press) do not elucidate the state of American psychiatry. Given Scull's title and introduction one might have expected commentary on such matters as practice patterns, successes, failures, and struggles of the 40 000 or so rank and file psychiatrists working in the United States, the high variance in outcomes among the people with mental illness for whom they care, and about the many people with mental illness in America who lack access to care because of substandard insurance, the criminalization of the mentally ill, and the maldistribution (and likely shortage) of mental health professionals. An appraisal of American psychiatry might have provided (within the length limits of such an opinion piece) selected cross-national comparisons such as latency to treatment of a first-psychotic episode or insight into premature mortality among the severely mentally ill (Hjorthoj, Sturup, McGrath, & Nordentoft, 2017), the latter an indictment of health care systems in all high-income countries that, pending further analysis, appears to reflect the lack of access to general medical care, homelessness, and incarceration more than psychiatric treatment.

Scull is drawn to careless or callous statements by well-known psychiatrists whatever their vintage. For example, he tells us that in 1968, early in the history of psychopharmacology, Nathan Kline was dismissive of tardive dyskinesia as a side effect of antipsychotic drugs (Scull, 2021). Kline was clearly wrong – unsurprisingly his view was widely rejected – but it is hard to see how an isolated statement in 1968 matters for the current state of American psychiatry. Second-generation antipsychotic drugs were developed largely to reduce motor side effects, including tardive dyskinesia and have largely succeeded in that regard, despite their own serious side effects and efficacy no greater than the older drugs (Lieberman et al., 2005). Even with respect to the DSM system, Scull misses the opportunity to ask deeper questions. How, for example, can a disease classification remain globally influential despite being produced by an organization that represents the interests of a single profession in single country – and that has its trustees vote on proposed changes to diagnostic criteria despite competing responsibilities to their membership.

Scull (2021) refers with approbation to comments by E. Fuller Torrey, who has argued that given pressing patient needs, expenditure on neuroscience and genetics represents a diversion from much-needed clinical research. It is disappointing that Scull, a historian, restates this point in his conclusion. Of course, intense impatience is warranted in light of the profound suffering, disability, and early deaths caused by mental illness even as decades pass without significant advances in treatment efficacy (Hyman, 2012). Lacking insight into mechanism, however, the alternative is clinical research based on hunches and guesswork. Given the serendipitous origins of current psychiatric therapeutics, it is important to distinguish guesswork from unbidden observations of the sort that led Alexander Fleming to penicillin, and that led the surgeon Henri Laborit to recommend chlorpromazine to the psychiatrists Delay and Deniker after his experience using it as a preanesthetic. Guesswork, in contrast, has failed psychiatry relentlessly. Pharmaceutical companies have disinvested in psychiatry for the lack of basic insight into the mechanisms of psychiatric disorders – the key platform for therapeutics research – in contrast to cancer, autoimmune disorders, and metabolism where the science is further advanced. Rejection of neurobiology and genetics would condemn psychiatry to reliance on luck.

Although an imperfect comparison to current psychiatry research, it is worth reflecting on Richard Nixon's War on Cancer formalized by the National Cancer Act of 1971. The War on Cancer unleashed a torrent of scientifically premature clinical trials that administered high doses of cytotoxic agents despite severe side effects, high death rates, and scant long-term therapeutic success. I am not suggesting that clinical trials of new agents in psychiatry would harm patients in the manner of cytotoxic chemotherapy. What I want to point to was far less visible than these clinical trials: supported by vast new financial and scientific infrastructure provided by the National Cancer Institute (NCI), cancer research embarked on a serious, long-term commitment to investigate the basic biology of cancer while developing a generation of scientists. Cancer research in the 1970s had enough traction on cancer biology to permit wise use of funds. These factors included direct access to human cancers through excisional biopsies and reasonable insight into disease mechanisms such as dysregulation of the cell cycle. Cancer biology remains difficult and feels painfully slow to those who lack effective treatments. However, these basic efforts were understood by the NCI and the scientific community to represent what might be called a ‘long game’. That long game in cancer began to pay off with a steep decline in cancer deaths beginning in 2001, 30 years after the National Cancer Act.

Psychiatric disorders are arguably far more challenging to study than cancer biology. In contrast to cancer, psychiatry research has received far less funding, has no access to relevant brain tissue and prior to modern large-scale genetics, lacked any clues to disease mechanisms. Yet, capitalizing on new 21st century technologies such as massively parallel DNA sequencing, powerful computational resources, and large databases that benefit all of medicine, such as the UK Biobank, in the first decade of this millennium, psychiatric research has found itself, at last, in a position to develop a scientifically well-grounded long game. The long game in psychiatry has begun organically, rather than signaled by a Presidential signature, with the advent of unbiased large-scale genetic studies grounded in global data-sharing collaborations such as the Psychiatric Genomics Consortium. Such efforts have expanded in recent years, inextricably engaged with mainstream biology; psychiatric research has, for example, been a key contributor to development of such molecular tools such as high-throughput single-cell analyses that, when applied to post-mortem human brains have provided critical information that facilitates the interpretation of complex polygenic signals that have emerged from genetics (Finucane et al., 2018; Macosko et al., 2015).

I worry that Scull has interpreted the genetic complexity and heterogeneity of psychiatric disorders to suggest that modern genetics is yet another intellectual *cul-de sac*. Since he quotes accurately from my work – and I do emphasize the complexity of disease-relevant genomic variation, the heterogeneity of psychiatric disorders, and the nondeterministic risk prediction yielded by such tools as polygenic scores (Hyman, 2021) – I apologize if in my zeal to espouse epistemic humility and rigorous commitment to a scientific long game I have inadvertently contributed to a nihilistic view. Ironically, I have underscored complexity not only because that is what nature has given us (Hyman 2010, 2021), but also to combat just the kinds of reductive and overconfident claims that Scull discredits at the beginning of his appraisal (Scull, 2021). In any case I do not think that the current era of genomics, neurobiology, large population-based datasets, and computational biology represents yet another false dawn. In the current era, psychiatry research is no longer isolated from the cutting edge of science, key results replicate, and many of the actors in this era represent a new generation of quantitatively sophisticated young scientists – too few of them psychiatrists – who a decade ago would never have considered a career studying schizophrenia or mood disorders. Prior to the advent of modern large-scale genomics there was simply too little traction on disease mechanisms to risk a career. These young scientists recognize that interpretation of polygenic signals and their integration with patterns of gene expression from thousands of cell types in human brains and ultimately with neural circuits, cognition, and behavior represent very hard problems but not impossible. Indeed, scientists in every field of medicine face similar challenges to some degree.

I was baffled by Scull's seemingly reductive, dualist views in his treatment of the term ‘biology’ in psychiatry. Properly understood, genetics and neurobiology cannot be set in opposition to societal influence, lived experience, or other environmental exposures. Lived experiences, such as grinding poverty, the experience of imprisonment, or other sources of traumatization are processed both consciously and unconsciously by our brains (and other organs as well) and may set the stage, in the context of genetic and developmental susceptibility, for such conditions as depression, anxiety, post-traumatic stress disorder, sleep disorders, hypertension, and metabolic derangements, among others. What neurobiology teaches is that ideas and lived experience are encoded and exert their effects on mental states, physiology, behavior, and risk of mental illness as a result of information processing and plasticity within synaptic networks. As non-dualists, neurobiologists recognize that these synaptic networks integrate experience, ideas, and trains of thought with effects of genes, developmental signals, drugs, and disease. The scientific paths to understanding our most complex organ in health and in illness will be long, multifarious, and characterized by many setbacks, but I would ask the hucksters and the critics alike: what alternatives do you propose?
